# Linking nutritional biochemistry and trophic ecology to health of adult female California sea lions in the Gulf of California

**DOI:** 10.1093/conphys/coaf056

**Published:** 2025-07-31

**Authors:** Ana I Montesinos-Laffont, Olga P García, Fernando R Elorriaga-Verplancken, Karina A Acevedo-Whitehouse

**Affiliations:** Unit for Basic and Applied Microbiology, Autonomous University of Querétaro, Carr. a Chichimequillas S/N, Ejido Bolaños. CP. 76140 Santiago de Querétaro, Mexico; Human Nutrition Research, Autonomous University of Querétaro, Av. de las Ciencias s/n, Nuevo Juriquilla, CP. 76230 Santiago de Querétaro, Mexico; Department of Fisheries and Marine Biology, Centro Interdisciplinario de Ciencias Marinas, Instituto Politecnico Nacional, Av. Instituto, Politécnico Nacional s/n, Playa Palo de Sta Rita, CP. 23096 La Paz, Mexico; Unit for Basic and Applied Microbiology, Autonomous University of Querétaro, Carr. a Chichimequillas S/N, Ejido Bolaños. CP. 76140 Santiago de Querétaro, Mexico

**Keywords:** California sea lion, Gulf of California, health, nutrition ecology, stable isotopes, *Zalophus* californianus, blood analytes

## Abstract

Our planet is experiencing sudden and unpredictable changes that affect most land and marine environments. We investigated blood analytes relevant to nutritional biochemistry and isotopic signatures of adult female California sea lions (CSL) from the Gulf of California, an area that has suffered changes in sea surface temperature in the past decades. During the 2016 and 2020 breeding seasons we collected fur, plasma and serum samples from apparently healthy adult female CSL (2016, *n* = 43; 2020, *n* = 12). We determined packed cell volume (PCV) and quantified 11 blood analytes directly or indirectly related to nutrition (albumin, cholesterol, triglycerides, glucose, total protein, globulin, creatinine, ferritin, iron, zinc and bilirubin). We also determined carbon and nitrogen isotopic signatures in the fur. Most analytes from 2020 were within the ranges reported for free-ranging CSL, while various analytes from 2016 deviated from reported ranges. Cholesterol, albumin, A:G ratio and zinc were higher in 2020, and glucose and total bilirubin were higher in 2016. Cholesterol and glucose varied across ecological regions. Isotopic values varied between sampling years, while trophic level and δ^15^N varied across regions. The δ^13^C values were related to blood glucose, while trophic level was related to cholesterol. These results may reflect dietary changes, as supported by isotopic signals. The variations in some of the blood analytes suggest short-term stressors or slight differences in sampling season, while others may reflect metabolic compensation of foraging effort, malnutrition or subclinical shifts in health. We generated reference data of the blood analytes for wild adult female CSL. By integrating clinical and ecological indicators, our approach offers a tool for early detection of subclinical metabolic and dietary shifts relevant to health and population viability. This is valuable for the conservation and adaptive population management of marine predators in rapidly changing ecosystems such as the Gulf of California.

## Graphical Abstract

**Graphical Abstract:**
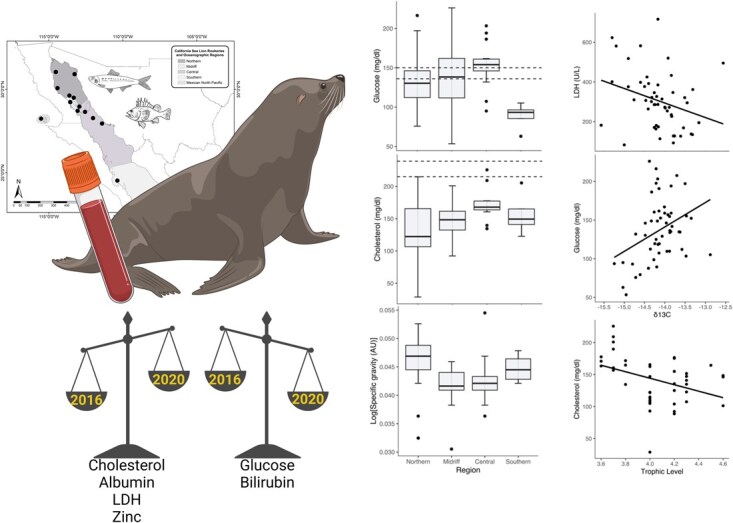

## Abbreviations

SST, sea surface temperatures;
A:G ratio, albumin to globulin ratio;
PCV, packed cell volume; δ^15^N, ^14^N/^15^N; δ^13^C, ^13^C/^12^C;
TL, trophic level;
GLMs, generalized linear models

## Introduction

Assessing the health of wild animal populations can be complex, particularly in the 21st century, a time characterized by unpredictable climatic variability, habitat degradation, increased contact with humans and domestic animals, urbanization, expanded military activity, increased energy production and resource extraction, among other stressors (Marzluff *et al*., 2001; Kennish, 2002; Foley *et al*., 2005; Dudgeon *et al*., 2006; Crain *et al*., 2009). The marine environment is not exempt from these changes, and it has faced many challenges in the past years. For instance, anomalously warm sea surface temperatures (SST), exacerbated by El Niño–Southern Oscillation (ENSO) events that have occurred in the Northeastern Pacific in the past decade, have affected many species throughout the food web, modifying available prey and leading to unusual mortality events (Elorriaga-Verplancken *et al*., 2022), population declines (Elorriaga-Verplancken *et al*., 2016; Rodríguez-Rafael *et al*., 2023) and physiological irregularities (DeRango *et al*., 2019) in top marine predators. These sudden and unexpected ecosystem changes can exert pressure at many levels, from the community to the molecular level, which is why a more holistic approach, such as that envisaged by Conservation Physiology (Cooke *et al*., 2013, 2020), has become urgent.

Nutrition ecology, the study of the relationships between feeding habits, dietary composition and digestive physiology (Raubenheimer *et al*., 2009) and trophic ecology are two subdisciplines that can further our understanding of wildlife health. This is because prey consumed by an individual leave an isotopic footprint that can be assessed using stable isotope ratios of nitrogen and carbon in different tissues, allowing to make inferences regarding trophic position/breadth and habitat use of consumers, respectively (Newsome *et al*., 2007), and the way in which an organism digests the prey, absorbs and uses nutrients to meet the cost of different physiological processes can be reflected in its blood analytes. In turn, nutrition ecology can help understand the links between animal physiology and ecological patterns and processes (Raubenheimer *et al*., 2009; Karasov *et al*., 2011; Simpson and Raubenheimer, 2012), including population dynamics (Foley and Cork, 1992; Raubenheimer *et al*., 2015). Alterations in prey availability, dietary changes and physiological demands can impact health, as is well known for human populations (Gimeno, 2003; Farré, 2005; FAO, 2007; Cena and Calder, 2020). In the context of rapid environmental changes, it is relevant to monitor blood chemistry of sentinel species, particularly as various analytes can allow us to detect biochemical deficiencies (Guerra Montemayor, 2010) or to identify suboptimal immune competence (Gibson, 2005; Lee and Nieman, 2007; Guerra Montemayor, 2010) before there are evident signs of disease (Grabow *et al*., 2024).

Since the 1990s, the Gulf of California has experienced a multi-decadal SST warming (Adame *et al*., 2020; López Martínez *et al*., 2023), which has been associated with a temporal reduction in the abundance of key species, including the Pacific sardine (*Sardinops sagax*) (Szteren *et al*., 2006), and a 65% decline in the size of the resident population of California sea lions, *Zalophus californianus* (hereafter, CSL), reaching ~15 291 (range: 11 861–20 316) in 2019, as a result of an apparent impact on its trophic dynamics (Adame *et al*., 2020). The Pacific sardine is one of the CSL’s preferred prey in this region (García-Rodríguez and Aurioles-Gamboa, 2004), and its reduced availability may lead to a dietary switch. This species has commonly been considered as an opportunistic predator that can adapt its diet based on availability and environmental conditions (Aurioles-Gamboa *et al*., 1984; Lowry *et al*., 1991; García-Rodríguez and Aurioles-Gamboa, 2004; Mellink and Romero-Saavedra, 2005; Villegas-Amtmann *et al*., 2011). CSL can also opt for prey of lower nutritional value, as has been reported for this species off the California coastline (McClatchie *et al*., 2016). Dietary switches during different life stages or due to changing climatic conditions have been described for other pinniped species (Hofmeyr *et al*., 2010; Szteren *et al*., 2018).

The SST warming of the Gulf of California is a good predictor of CSL demography, as it accounts for up to 93% of the population variability (Adame *et al*., 2020). Abnormally high SST are also related to shifts in maternal foraging in this species (Elorriaga-Verplancken *et al*., 2016; Cruz-Vallejo *et al*., 2024) and these have been linked to suboptimal pup immune responses (Banuet-Martínez *et al*., 2017) and alterations in neonatal blood physiology (Flores-Morán *et al*., 2017). However, to date, there are few published studies on the impact that climatic and ecological changes can exert on wider aspects of physiology and health of adult CSL. Being a sentinel species of the coastal marine ecosystem (Le Boeuf *et al*., 2002), it is relevant to understand the resilience of their physiology in the context of ecological changes, as this can be informative about their general health. Here, we focus on how trophic ecology and nutritional biochemistry of adult female CSL vary across the ecological regions of the Gulf of California during two sampling seasons, 2016, a year with relatively high SST (1.73°C on average), and 2020, a year with relatively low SST (0.84°C on average).

## Materials and Methods

### Sampling locations

**Figure 1:**
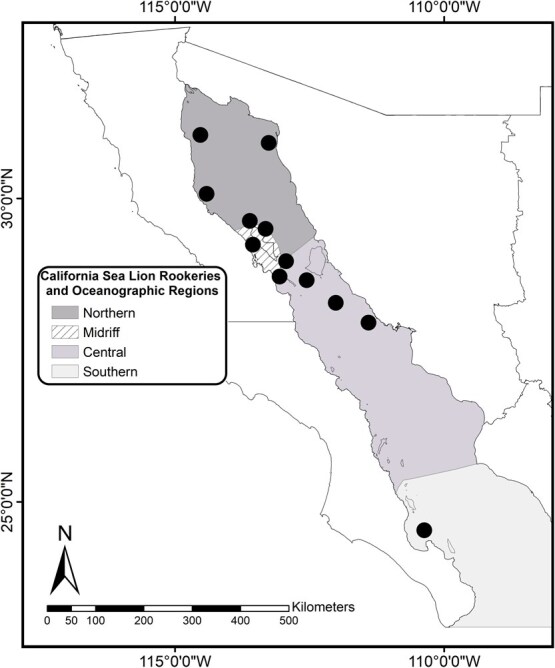
Study site in the Gulf of California. The map shows the breeding rookeries and the ecological regions.

Within the Gulf of California there are 13 rookeries (Fig. 1) distributed along the four ecological regions identified (Lluch-Cota, 2004; Lara-Lara *et al*., 2008; Szteren and Aurioles-Gamboa, 2011): three in the Northern region (Rocas Consag, Isla San Jorge and Isla Lobos), four in the Midriff (Granito, Los Cantiles, Los Machos and El Partido), four in the Central region (El Rasito, San Esteban, San Pedro Martir and San Pedro Nolasco), and two in the Southern region (Los Islotes and Farallon de San Ignacio) (Szteren and Aurioles-Gamboa, 2011). We conducted field expeditions to sample adult female CSL in 12 of these breeding rookeries (all except Farallón de San Ignacio; see Fig. 1) within these ecological regions during 2016 (15–30 July) and 2020 (15–30 August).

### Sampling procedures

Adult female CSL (43 during the 2016 expedition and 12 during the 2020 expedition) were captured using hoop nets and were anaesthetised using inhaled isoflurane (2%, delivered at 2 l/min via a facemask) connected to a portable anaesthesia machine (Transport 5000 plus, DRE) or were manually restrained, depending on the level of stress displayed by the female in turn (i.e. struggling during handling). The decision for physical or chemical containment was made by the veterinarian to ensure the well-being of the sea lions. Details on the containment method used can be seen in Table 1. Each sea lion was evaluated to determine their general health condition (assessed by a veterinarian who examined their blubber layer thickness visually by ensuring the ribcage and hipbones were not visible, pelage and skin condition, evidence of trauma and colour of mucosae) and reproductive status (i.e. evidence of having given birth to a pup in that season; determined by examination of the cervix or by direct observation of nursing immediately before capture). Vital signs (palpebral reflex, eye position, iris appearance, respiratory frequency and capillary refill) were monitored throughout chemical immobilization, which lasted between 10 and 12 min. Handling time was similar when using manual restraint. All procedures were approved by the Bioethics Committee of the Autonomous University of Querétaro (Mexico) and were conducted under permits (SGPA/DGVS/09004/15 and SGPA/DGVS/11794/19) by the Secretaría de Medio Ambiente y Recursos Naturales through the Dirección General de Vida Silvestre in Mexico.

**Table 1:** Containment methods used during sampling of the adult female CSL and of the quality of the plasma and serum samples obtained for each cohort

Year	CSL sampled	CSL manually restrained	CSL anesthetized	Haemolysed plasma	Haemolysed serum	Lipemic plasma	Lipemic serum
2016	43	25	18	2	20	9	8
2020	12	12	0	0	3	3	3

For each sea lion, a fur sample (~20 mg) was collected from the rump, using scissors that were cleaned between sampling, and was kept in a paper envelope. Blood samples were obtained from the caudal gluteal vein using 20G needles and sterile vacuum tubes (BD Vacutainer). A 5-ml sample was collected in a sterile vacuum tube with ethylenediaminetetraacetic acid (EDTA), and a 7-ml sample was collected in a sterile vacuum tube with no preservative. Blood samples were kept upright in a 4°C cooler, protected from the sun and were processed within 4 h of collection. Packed cell volume (PCV) was determined on-site using a microhaematocrit centrifuge (CEN-6T, Zeigen). Blood samples were centrifuged for 10 min using a clinical centrifuge (Clay Adams compact II, Daigger Scientific) with a fixed speed of 3200 rpm. Using a sterile Pasteur pipette, EDTA plasma or serum were separated from the blood tubes and collected in sterile 1.5-ml cryotubes. Plasma and serum samples were immediately frozen in liquid nitrogen and then transferred to a −80°C freezer in the laboratory where they remained until biochemical analyses were performed. Plasma colour was assessed for each sample. All analyses were carried out in August 2021, meaning that serum and plasma samples collected in 2016 had been stored for 5 years and samples collected in 2020 had been stored for 1 year. Variation due to storage is considered of little to no significance for all other blood analytes provided the temperature of the freezer remains constant (Thoresen *et al*., 1992; Thoresen *et al*., 1995; Reynolds *et al*., 2006; Cray *et al*., 2009; Peng *et al*., 2010; Schlake *et al*., 2023).

### Biochemical analyses

We quantified 11 blood analytes that are directly or indirectly relevant for assessing nutritional health. These were: albumin, cholesterol, triglycerides, glucose, total protein, globulin, bilirubin, creatinine, ferritin, iron and zinc. We also calculated the albumin to globulin ratio (A:G). EDTA plasma was used for the analyses of glucose, albumin, iron, total protein, total cholesterol, triglycerides, bilirubin and creatinine, and serum was used to quantify zinc and ferritin.

Glucose, albumin, iron, total protein, total cholesterol, triglycerides, bilirubin and creatinine were quantified in the EDTA plasma samples using commercial kits in an automated biochemistry analyser Spin 120 (Spinreact, Girona, Spain), following the manufacturer’s protocols (Spinreact; glucose, kit 41 013; albumin, kit 1 001 020; iron, kit 1 001 247; total protein, kit 1 001 291; cholesterol, kit 41 021; triglycerides, kit 41 033; bilirubin, kit 1 001 046; creatinine, kit 1 001 113). For quality control and calibration, we used Spintrol H calibrator (1002012), Spintrol H Normal (1002120) and Spintrol H Pathological (1002210) and quantified the values for each analyte in the EDTA plasma samples. The use of EDTA does not affect the quantitation of these blood analytes (Ahn *et al*., 2016; Ford *et al*., 2022). Ferritin and zinc were analysed using serum samples*.* To quantify ferritin, we ran enzyme-linked immunosorbent assays (ELISAs) on the serum samples using Abcam (ab108837) and read them in a Multiskan Ascent (Thermo Electron Corporation, USA). This technique has been validated using human blood (Liu *et al*., 2017; Choi *et al*., 2019). Zinc was quantified in an Analyst 700 Atomic Absorption Spectrometer (AAS, Perkin Elmer, USA) using 1000 mg/l of N9300178 (Perkin Elmer) as a calibrator, as well as quality control serum (Randox), level 2 control serum (HN1530) and level 3 control serum (HE1532). Globulin was determined by subtracting albumin from total protein for each sample (Duvall *et al*., 2023).

### Stable isotope analysis

All fur samples were processed at the Chemistry Lab of the Centro Interdisciplinario de Ciencias Marinas (CICIMAR-IPN) in La Paz, Baja California Sur, Mexico. Briefly, samples were washed with a 1:1 chloroform–methanol solution to remove impurities and a second wash was done with distilled water. Hairs were cut and homogenized using an agate mortar in a laminar flow hood. Each sample was weighed using a precision microbalance and 0.8–1.2 mg were placed in tin capsules that were sent to the Stable Isotopes Laboratory at the University of New Mexico. Quantitation of ^14^N/^15^N (δ^15^N) and ^13^C/^12^C (δ^13^C) was conducted by continuous-flow Isotope-Ratio Mass Spectrometry in a Carlo Erba 1108 elemental analyser coupled to a ThermoFinnigan Delta Plus XP isotope ratio mass spectrometer. Analytical precision was ~0.2‰ for both stable isotope ratios. The isotopic compositions of the samples were determined based on standards Vienna Pee Dee Belemnite (VPBD) for δ^13^C and atmospheric nitrogen for δ^15^N.

To establish the basal trophic level (TL) for each sampling region, zooplankton samples were collected during the 2016 field expedition near Rocas Consag, Isla Lobos, Los Cantiles, Los Machos and El Partido rookeries. For the southern zones, data were taken from Whitehead *et al*. (2018). The zooplankton samples were processed and analysed as described above for the fur samples.

Data on δ^13^C and δ^15^N were calculated for each sample according to the following formula by DeNiro and y Epstein S. (1978):


$$ {\mathrm{\delta}}^{15}\mathrm{N}\ \mathrm{or}\ {\mathrm{\delta}}^{13}\mathrm{C}=1000\ \left[\left(\mathrm{Rsample}/\mathrm{Rstandar}\right)-1\right]. $$

The delta units (δX) are expressed in parts per thousand (‰). Each analysis shows the difference between the ^15^N or ^13^C reference sample and the ^15^N or ^13^C evaluated sample. To determine the TL in each rookery, we used the algorithm proposed by Post (2002):


$$ \mathrm{TL}=\frac{\left({\delta}^{15}\mathrm{N}\ \mathrm{consumer}-{\delta}^{15}\mathrm{N}\ \mathrm{base}\right)}{3.4}+2 $$

where δ^15^N consumer was the value obtained for each fur sample, δ^15^N base was the value obtained for zooplankton at the relevant sampling site, and 3.4 is the trophic fractionation of δ^15^N across the food chain (Minagawa and Wada, 1984). For those rookeries for which zooplankton samples were not collected, we used the values for samples collected from the nearest rookeries. We were unable to calculate TL for samples collected in 2020 as we did not have any zooplankton sample to use as the base value.

**Table 2:** Mean and 95% CI of blood analytes of adult female CSL in the Gulf of California

Analyte	2016	2020	Study #1^a^	Study #2^b^
Albumin (g/dl)	3.4^**^ (3.3–3.5) *n* = 43	3.7 (3.6–3.8) *n* = 12	3.8 (3.8–3.9) *n* = 66	3.2 (3.1–3.3)
Globulin (g/dl)	4.73 (4.20–5.26) *n* = 43	4.44 (4.09–4.79) *n* = 12	ND	ND
A:G ratio	0.74^*^ (0.66–0.81) *n* = 43	0.85 (0.77–0.94) *n* = 12	ND	ND
Total protein (g/dl)	8.1 (7.9–8.5) *n* = 43	8.1 (7.8–8.5) *n* = 12	8.0 (7.7–8.2) *n* = 66	7.8 (7.7–7.9)
Cholesterol (mg/dl)	141.6^***^ (134.1–156.2) *n* = 43	176.9 (163.3–190.5) *n* = 12	ND	227.0 (213.0–239.0)
Triglycerides (mg/dl)	50.3 (42.9–60.6) *n* = 42	45.4 (35.6–55.3) *n* = 12	112.0 (94.3–129.7) *n* = 67	45.0 (38.0–52.0)
Glucose (mg/dl)	143.3^*^ (132.6–154.8) *n* = 43	114.1 (91.8–136.3) *n* = 12	113.0 (106.4–119.6) *n* = 80	143.0 (136.0–150.0)
Bilirubin (mg/dl)	0.58^*^ (0.51–0.65) *n* = 43	0.44 (0.35–0.52) *n* = 12	0.20 (0.05–0.35) *n* = 28	0.20 (0.10–0.30)
Creatinine (mg/dl)	1.5 (0.9–2.4) *n* = 43	1.6 (1.5–1.8) *n* = 12	0.7 (0.7–0.8) *n* = 60	1.0 (0.9–1.1)
				
Ferritin (ng/ml)	23.9 (0.7–137.1) *n* = 40	24.5 (0–47.7) *n* = 12	ND	ND
Iron (μg/dl)	189.3 (172.2–208.4) *n* = 42	164.8 (130.3–199.4) *n* = 12	ND	ND
Zinc (mg/l)	0.49^*^ (0.45–0.53) *n* = 36	0.58 (0.51–0.64) *n* = 12	ND	ND
PCV (%)	54.82 (52.89–56.53) *n* = 36	55.04 (53.32–56.75) *n* = 12	46.00 (45.07–46.93) *n* = 93	44.31^c^ (42.63–45.9)
				

For comparison, we include these mean and 96% CI of CSL from the California coastline (USA) from two separate studies. Except for ferritin and zinc, which were measured in serum, all blood analytes were measured in plasma.

ND, not determined. When values differed between 2016 and 2020, significance is shown as ^*^P < 0.05, ^**^P ≤ 0.01, ^***^P ≤ 0.001.

a Values were calculated as pooled results for adult and juvenile females and males (Roletto, 1993)

b Values were calculated as pooled results for males and females of all age classes, and include only three adult females (Williams, 2013).

c Value reported is haematocrit, estimated by Williams, 2013 as HCT = (packed cell volume × red blood cell count) / 10

### Statistical analyses

We first explored our dataset graphically to establish the spread and distribution of the data, identify outliers and examine the relationships among variables. Following an initial examination distribution and assessment of kurtosis and skewness of the data, we used Cullen and Frey graphs for each of the blood analytes to determine their distribution. Globulin values and A:G ratios were normally distributed; albumin and total protein had a lognormal distribution, and the rest of the biochemical variables had a beta distribution. To account for potential variations due to differences in handling of the CSL prior to challenging our working hypothesis, we investigated whether any of the blood analytes varied according to handling (manually restrained vs anaesthesia). We built independent generalized linear models (GLMs) to challenge the following hypothesis: (i) blood analytes associated with nutritional status vary between years, (ii) each of the blood analytes varies among ecological regions and (iii) feeding habits (assessed by δ^13^C and TL) explain differences in the values of blood analytes. Response variables that had a beta distribution were transformed by dividing the variable by its maximum value to have all values between 0 and 1 and were then modelled with a quasibinomial error distribution and logit link. Both variables with a lognormal distribution were log-transformed and were modelled with a Gaussian error distribution. To correct for multiple testing, we used Hochberg procedures on each hypothesis (Roback and Askins, 2005). We pooled the 2016 and 2020 data for each blood analyte. We identified outliers using a Grubbs test and used the package ‘referenceIntervals’ (Finnegan, 2024) to generate the reference intervals in accordance with the guidelines proposed by Friedrichs *et al*. (2012). All analyses were run in R v4.1.3 (R Core Team, 2016).

## Results

All CSL that were included in the study were adult females deemed to be clinically healthy following examination by a veterinarian, and they all had given birth to a pup during that year’s pupping season, as determined by direct observation of nursing prior to capture or examination of the cervix. As 42% of the samples showed evidence of mild (degree: +1) haemolysis and 20% of the samples had mild (degree: +1) lipemia (see Table 1), each sample type (serum or plasma) analysed included a sample blank to eliminate noise due to the sample’s coloration. The containment method used (manual vs anaesthesia) did not affect any of the blood analytes (*P* > 0.05 for all pairwise comparisons).

**Figure 2:**
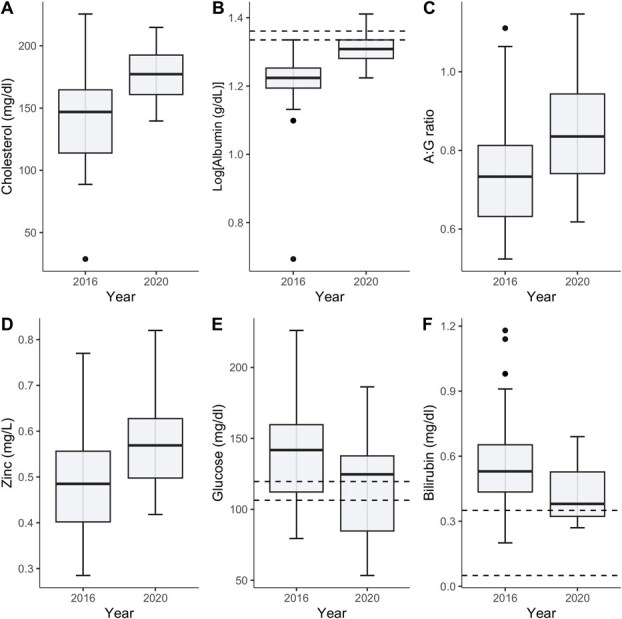
Blood analytes of adult female CSL sampled in 2016 and in 2020 in the Gulf of California. A) Cholesterol, B) Albumin, C) A:G ratio, D) Zinc, E) Glucose, F) Bilirubin. Dashed lines show the 95% confidence interval (CI) from a previous study of free-ranging CSL from US waters and corresponds to pooled results for female and males of juvenile and adult age classes (Roletto, 1993; see Table 2). A:G ratio, Albumin to globulin ratio.

Most, but not all, blood analytes in 2020 were within the ranges reported previously for free-ranging adult CSL (Table 2). Eight of the analytes varied significantly between sampling years. These were cholesterol (GLM; *t* = 3.07, *P* = 0.001; Fig. 2A), albumin (GLM; F_1,53_ = 10.63, *P* = 0.002; Fig. 2B), A:G ratio (GLM; F_1,53_ = 6.34, *P* = 0.015; Fig. 2C), zinc (GLM; *t* = −2.02, *P* = 0.02; Fig. 2D), glucose (GLM; *t* = 2.36, *P* = 0.02; Fig. 2E), bilirubin (GLM; *t* = 2.09, *P* = 0.04; Fig. 2F); the first four being significantly higher in 2020, and the latter two being significantly higher in 2016. The reference intervals of the pooled data for each blood analyte can be seen in Table 3.

**Table 3:** Reference intervals of blood analytes of free-ranging apparently healthy adult female CSL in the Gulf of California

Blood analyte	*n*	Mean ± SD	Median	Min–Max	RI	90% CI LRL	90% CI URL	Dist.	Method	*P-*value
Albumin (g/dl)	54	3.4 ± 1.0	3.4	3.0–4.1	3.0–3.9	2.9–3.1	3.8–3.9	NG	T/P	1.2e^−05^
Globulin (g/dl)	54	4.7 ± 0.7	4.7	3.1–6.4	3.0–6.2	2.8–3.4	5.9–6.5	G	P	0.8
A:G ratio	55	0.8 ± 0.1	0.7	0.5–1.1	0.4–1.0	0.4–0.5	0.9–1.1	G	P	0.05
Total protein (g/dl)	54	8.1 ± 1.1	8.3	6.4–9.9	6.6–9.9	6.4–6.9	9.5–10.3	NG	T/P	0.7e^−03^
Cholesterol (mg/dl)	55	151.5 ± 33.9	153.3	88.7–225.6	75.8–222.7	61.6–90.0	208.5–236.9	G	P	0.3
Triglycerides (mg/dl)	53	47.4 ± 24.0	44.7	9.5–118.4	10.3–115.1	0.0–11.1^a^	111.8–135.0^a^	NG	NP	0.2e^−03^
Glucose (mg/dl)	55	136.9 ± 39.2	134.8	53.4–226.1	60.0–213.8	45.1–74.8	198.9–228.7	G	P	0.6
Bilirubin (mg/dl)	52	0.5 ± 0.2	0.5	0.2–1.1	0.1–0.9	0.04–0.2	0.8–1.0	NG	T/P	0.1e^−02^
Creatinine (mg/dl)	55	1.5 ± 0.3	1.6	0.9–2.1	0.8–2.1	0.7–1.0	2.0–2.3	G	P	0.2
Ferritin (ng/ml)	51	21.6 ± 31.4	9.3	0.7–132.0	0.7–128.1	0.7–0.7^a^	124.3–157.3 ^a^	NG	NP	6.9e^−10^
Iron (μg/dl)	53	180.2 ± 54.4	168.0	93.0–299.0	72.9–284.0	52.9–93.2	264.0–304.6	NG	T/P	0.4e^−02^
Zinc (mg/l)	48	0.5 ± 0.2	0.5	0.2–0.8	0.2–0.7	0.2–0.3	0.7–0.8	G	P	0.6
PCV (%)	50	54.2 ± 1.08	54.8	42.1–69.2	48.6–60.9	47.5–49.6	59.5–62.2	NG	T/P	0.1e^−02^

The reference interval was generated using the package ‘referenceIntervals’ in RStudio v1.3.1093. *n*, number; SD, standard deviation; Min, minimum; Max, maximum; RI, reference intervals; CI, confidence interval; LRL, lower reference limits; URL, upper reference limits; Dist, Distribution; G, Gaussian; NG, non-Gaussian; T, transformed; P, parametric; NP, non-parametric.

a Sample size too small for non-parametric CIs, bootstrapping instead.

**Figure 3:**
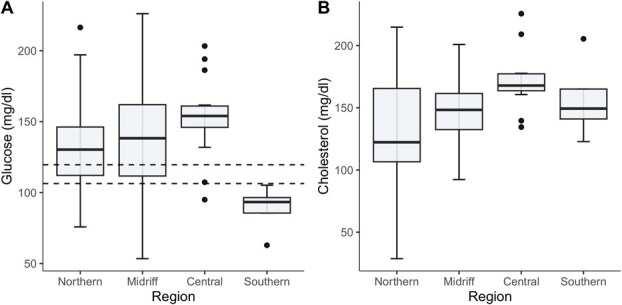
Regional variation in blood analytes of adult female CSL from the Gulf of California. (A) Glucose, (B) Cholesterol. Dashed lines show the 95% CI from a previous study of free-ranging CSL from US waters and corresponds to pooled results for female and males of juvenile and adult age classes (Roletto, 1993; see Table 2). Blood analytes were measured using plasma samples.

When investigating the variation in the blood analytes by ecological region, we only detected significant differences for cholesterol and glucose (see models in Table 4). Namely, considering differences between sampling years, the highest values of cholesterol and glucose were observed in the central region, while the lowest glucose values were found in the southern region (Fig. 3A) and the lowest cholesterol values were found in the northern region (Fig. 3B).

**Table 4:** Model outputs for blood analytes of CSL that varied in terms of ecological region and year

Term	*df*	Deviance resid.	*df*	Resid. dev	*P*-value
Cholesterol (beta distribution; data was divided by maximum value and modelled using a quasibinomial distribution) Model: glm(Cholesterol~Region^*^Year)					
Null			54	7.2664	
Region	3	1.0434	51	7.2664	0.0030
Year	1	1.0073	50	6.2230	0.0002
Region:Year	3	1.3057	47	5.2157	0.0006
Glucose (beta distribution; data was divided by maximum value and modelled using a quasibinomial distribution) Model: glm(Glucose~Region^*^Year)					
Null			54	7.6456	
Region	3	1.11868	51	6.5270	0.0159
Year	1	0.68453	50	5.8424	0.0119
Region:Year	3	0.06221	47	5.7802	0.9022

*df*, degrees of freedom; Resid, residual; Dev, deviance

**Figure 4:**
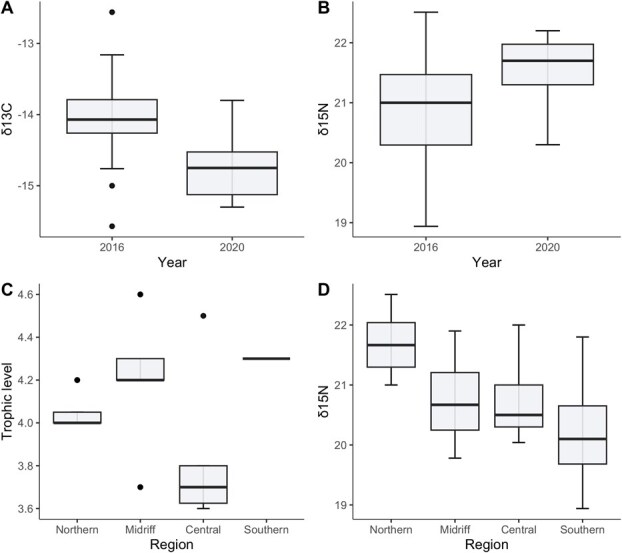
Trophic signals of fur samples from adult female CSL sampled in 2016 and in 2020 in the Gulf of California. (A) δ^13^C values varied between years, (B) δ^15^N values varied between years, (C) TL varied across regions, (D) δ^15^N values varied across regions.

**Figure 5:**
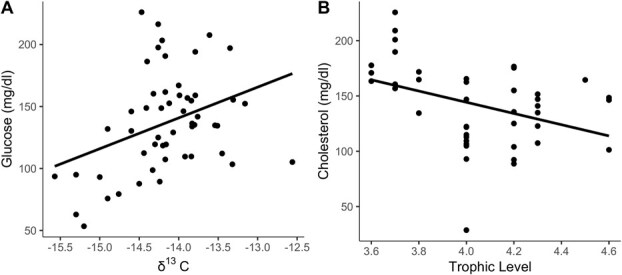
Relationship between trophic signals of fur samples and blood analytes of adult female CSL from the Gulf of California. (A) Glucose concentration varies proportionally to δ^13^C values. (B) Cholesterol concentration varies inversely with TL. Blood analytes were measured using plasma samples.

δ^13^C and δ^15^N values varied between sampling years (δ^13^C: GLM, F_1,45_ = 15.96, *P* = 0.0002, Fig. 4A; δ^15^N: GLM, F_1,50_ = 4.31, *P* = 0.043, Fig. 4B). Both TL and δ^15^N varied significantly across ecological regions (TL: GLM, F_3,36_ = 6.27, *P* = 0.002, Fig. 4C; δ^15^N: GLM, F_3,49_ = 12.80, *P* = 2.68x10^−6^, Fig. 4D), while δ^13^C values did not (*P* > 0.05). Considering sampling year, δ^13^C significantly explained individual variation in blood glucose, where sea lions with a higher δ^13^C value had higher glucose (GLM, *t* = 3.00, *P* = 0.003; Fig. 5A). Sea lions with a higher TL had lower blood cholesterol, even when considering differences in cholesterol between ecological regions (GLM, *t* = 2.01, *P* = 0.05; Fig. 5B).

## Discussion

Nutritional ecology explores the relationship between ecological variables, feeding behaviours and individual biochemical markers, providing insight into an organism’s health and population dynamics (Clements *et al*., 2009; Raubenheimer *et al*., 2009; Lihoreau *et al*., 2015; Raubenheimer and Simpson, 2018). Here, we assessed the nutritional biochemistry of free-ranging adult female CSL from different ecological regions in the Gulf of California, focusing on blood variables and their isotopic niche as reflections of their foraging habits.

The interannual differences in SST in the study region were substantial, with significantly higher temperatures recorded in 2016 compared to 2020 (an average 12-month anomaly almost 1°C higher in 2016, and nearly 2°C higher during some months; López Martínez *et al*., 2023; NOAA, 2024; see Supplementary Fig. 1). These thermal anomalies are known to influence prey availability and distribution, triggering dietary shifts in marine predators (McClatchie *et al*., 2016; Robinson *et al*., 2018). Indeed, our stable isotope analysis revealed variation in CSL foraging strategies and trophic positions among regions and between years, consistent with differential prey use. These foraging adaptations may reflect ecosystem changes (Adame *et al*., 2020), such as the longstanding collapse of sardine populations since the late 1980s (Nevárez-Martinez *et al*., 2001), coupled with localized increases in other small pelagic fish (García-Rodríguez and Aurioles-Gamboa, 2004; Espinosa de los Reyes Ayala, 2007). Together, these findings suggest that the adult females that we studied may have adjusted their foraging strategies in response to environmental and prey variability—a central tenet of nutritional ecology.

The trends of several of the CSL blood analytes we examined could reflect such dietary shifts. Cholesterol concentrations were significantly higher in 2020, a year with cooler SST and evidence of more offshore foraging, inferred from the lower δ^13^C values we recorded (Burton and Koch, 1999; Kuhn and Costa, 2014; Rosas-Hernández *et al*., 2018). Cholesterol is mainly obtained by the diet (Chapman, 1980; Formanowicz *et al*., 2022) and CSL are generalist predators whose diet varies depending on the ecosystem they inhabit and the availability of prey (Antonelis Jr and Fiscus, 1980). Warmer temperatures may have constrained foraging efficiency or shifted prey availability towards lower lipid prey, which is inferred by the relationship between cholesterol and TL, as evaluated by isotopic signal. High trophic-level prey like squid (*Leachia* sp.) and midshipman fish (*Porichthys* sp.)—common in more offshore zones—are lower in cholesterol than small pelagic fish such as anchovies and sardines (Childs *et al*., 1990), potentially explaining the higher values found in 2020, as supported by the isotopic signatures that year.

Further supporting this ecological–nutritional cascade, zinc concentrations also varied significantly between years, with higher values in 2020. As a dietary micronutrient (Rubio *et al*., 2007), zinc intake is tightly linked to prey composition. For example, sardines provide 0.7 mg of zinc per 200 g, compared to 1.7 mg per 200 g in squid (Moreiras *et al*., 2013). The difference in mean zinc blood concentrations was 0.1 mg/ml between 2016 and 2020, and some of the sea lions sampled in 2020 exceeded 0.8 mg/ml. The parallel increases in cholesterol and zinc concentrations in 2020 suggest that foraging on higher TL, offshore prey may have delivered greater nutritional value or at least altered micronutrient intake, even under potentially more demanding foraging conditions. Thus, cholesterol and zinc appear to be useful indicators of trophic ecology and nutritional status of CSL, though we caution against definitive causal claims without controlled dietary data.

Glucose concentrations were higher in 2016, particularly in the southern region. As glucose is a highly variable marker influenced by activity, diet, and stress (Bennett *et al*., 2017; Hantzidiamantis *et al*., 2022), several factors need to be considered in the interpretation of this difference. With a field study such as ours, which sampled adult female CSL, sample collection could not be controlled for differences in time elapsed since last foraging trip or nursing, variations in physical activities prior to capture and in the effects from capture and handling (Cherel *et al*., 1988; Romero and Beattie, 2022). However, the latter is less likely since there was no statistical effect. Additional considerations include differences in physiological stress associated with higher SST in 2016 or effects from sampling time point of animal capture in 2020 as it was later during the lactation period, when blood glucose is considered lower due to reduced maternal provisioning of resources (Bennett *et al*., 2017). This result may reflect that sea lions that are feeding further from the coast (inferred from more negative δ^13^C values) are investing more glucose to sustain their physical activity.

Albumin concentrations also varied between years, being significantly higher in 2020. This protein is influenced by both dietary protein intake and health status, with deviations from the normal range reflecting changes in diet or body condition (Vail *et al*., 2022) although together with other blood analytes may also be indicative of acute or chronic disorders (Ridgway, 1972; Browne, 1995; Soeters *et al*., 2019). Low albumin values are often associated with malnutrition, inflammation or infectious disease (Bossart and Dierauf, 1990; Don and Kaysen, 2004; Guerra Montemayor, 2010; Vail *et al*., 2022). The lower albumin values in 2016 could thus be indicative of reduced protein intake or physiological stress, while the higher values in 2020 may reflect better nutritional status—again potentially tied to prey composition and availability. The higher bilirubin concentrations in 2016 may indicate differences in feeding or fasting prior to sample collection, as no evidence of liver dysfunction was apparent in any of the study animals (Acevedo-Whitehouse, 2019).

Iron and ferritin levels of the sea lions remained stable across years and regions, regardless of changes in diet or SST. This may suggest that either the new prey had similar iron content, intake was adjusted to compensate, or dietary diversification balanced micronutrient intake. Alternatively, adult sea lions may tightly regulate iron absorption and storage, using internal reserves if needed, as can occur in other carnivores (Waldvogel-Abramowski *et al*., 2014). While this regulatory mechanism remains unstudied in pinnipeds, our findings underscore the crucial role of iron in energy metabolism and cellular function (Moreira *et al*., 2020).

Finally, not all analyte values fell within previously published reference ranges (Williams, 2013). Namely, the reported concentrations of cholesterol of free-ranging apparently healthy CSL from California were higher (95% CI = 213–239 mg/dl) than the highest concentrations we detected in our study (226 mg/dl) and only five samples (two from 2016 and three from 2020) were within that reported range (Williams, 2013). As there is no other published study to report blood cholesterol concentrations for free-ranging CSL, it is difficult to make direct comparisons with our findings as the data reported pooled all age classes and sexes (including only three adult females). Blood glucose concentrations were also wider ranging than those reported previously for healthy free-ranging CSL in California (Roletto, 1993; Williams, 2013). Considering that all the individuals we sampled were deemed to be in good health, we suggest that the normal ranges of blood cholesterol and glucose in adult female CSL are wider than previously reported for the species, and that the wide range in cholesterol values reflect dietary composition, metabolic differences and ecosystem variations. However, it is important to note that published CSL clinical reference values often include different age classes and both sexes, limiting direct comparisons. Our data support the need to establish age- and sex-specific baselines, particularly within ecological contexts.

To conclude, when considered collectively, our results underscore the value of integrating stable isotope ecology with blood biochemistry to assess population-level health in free-ranging pinnipeds. The observed patterns in analytes suggest that nutritional consequences of ecosystem variability are detectable using simple, low-cost assays. While we cannot assign definitive causality, the convergence of environmental (SST), ecological (isotopic signatures) and physiological (biochemical) data form a coherent picture: there is evidence that adult female CSL respond to ecosystem changes with foraging shifts that result in measurable physiological changes.

Our study provides a framework for investigating subclinical health changes in response to ecological dynamics, offering an important tool for early detection of population stressors. Given the increasing environmental variability along the CSL range (Nevárez-Martinez *et al*., 2001; Lluch-Cota, 2004; Adame *et al*., 2020), nutritional indicators that are sensitive to environmental factors can be a valuable tool for identifying subclinical metabolic differences and dietary shifts in CSL, informing proactive conservation strategies and adaptive population management in the face of sudden ecosystem changes.

## Supplementary Material

Web_Material_coaf056

## Data Availability

The datasets used for this study are available on ResearchGate (https://www.researchgate.net/publication/388621702_Montesinos-Laffont_et_al_CSL_blood_markers_trophic_ecology) and the R code used for analyses is available upon request to the corresponding author.
