# Supplementary material for: Linking nutritional biochemistry and trophic ecology to health of adult female California sea lions in the Gulf of California
Source: Conserv Physiol. 2025 Jul 31;13(1):coaf056. doi: 10.1093/conphys/coaf056 (PMC12362241; doi:10.1093/conphys/coaf056)
Supplement: Web_Material_coaf056 [file web_material_coaf056.zip › Montesinos-Laffont et al_Revision2_Supplementary material.pdf]

Supplementary material

Montesinos-Laffont et al. Linking Nutritional Biochemistry and Trophic Ecology to Health of Adult Female California Sea Lions in the Gulf of California

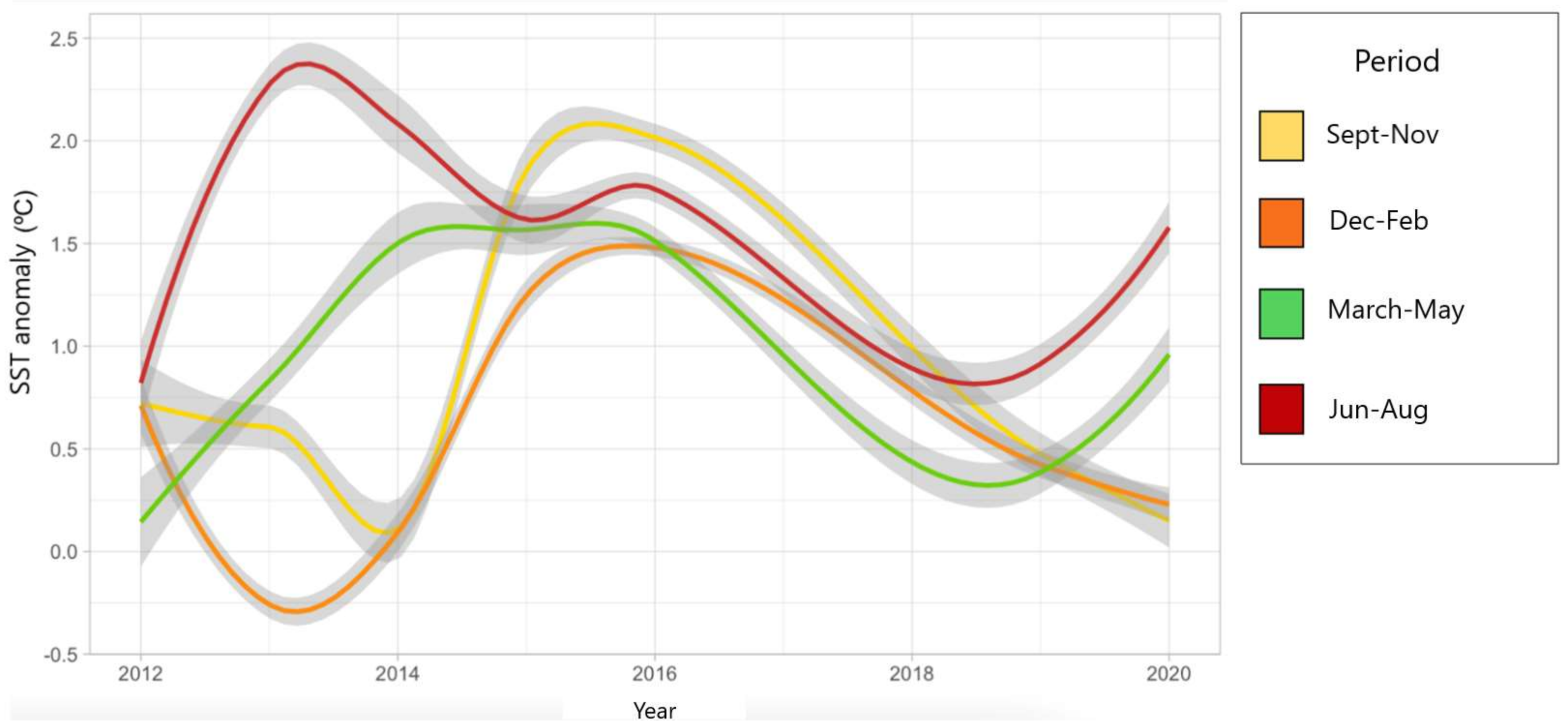

Supplementary Figure 1. Sea surface temperature (SST) anomaly in the Gulf of California. Figure generated with data from NOAA ERDDAP (https://www.ncei.noaa.gov/erddap/index.html) using the following search criteria: "SST, Daily Optimum Interpolation (OI), AVHRR Only, Version 2.1, Final, Global, 0.25°, 1981-present, Lon+/-180".
