# Supplementary figures and images for: Linking nutritional biochemistry and trophic ecology to health of adult female California sea lions in the Gulf of California
Source: Conserv Physiol. 2025 Jul 31;13(1):coaf056. doi: 10.1093/conphys/coaf056 (PMC12362241; doi:10.1093/conphys/coaf056)

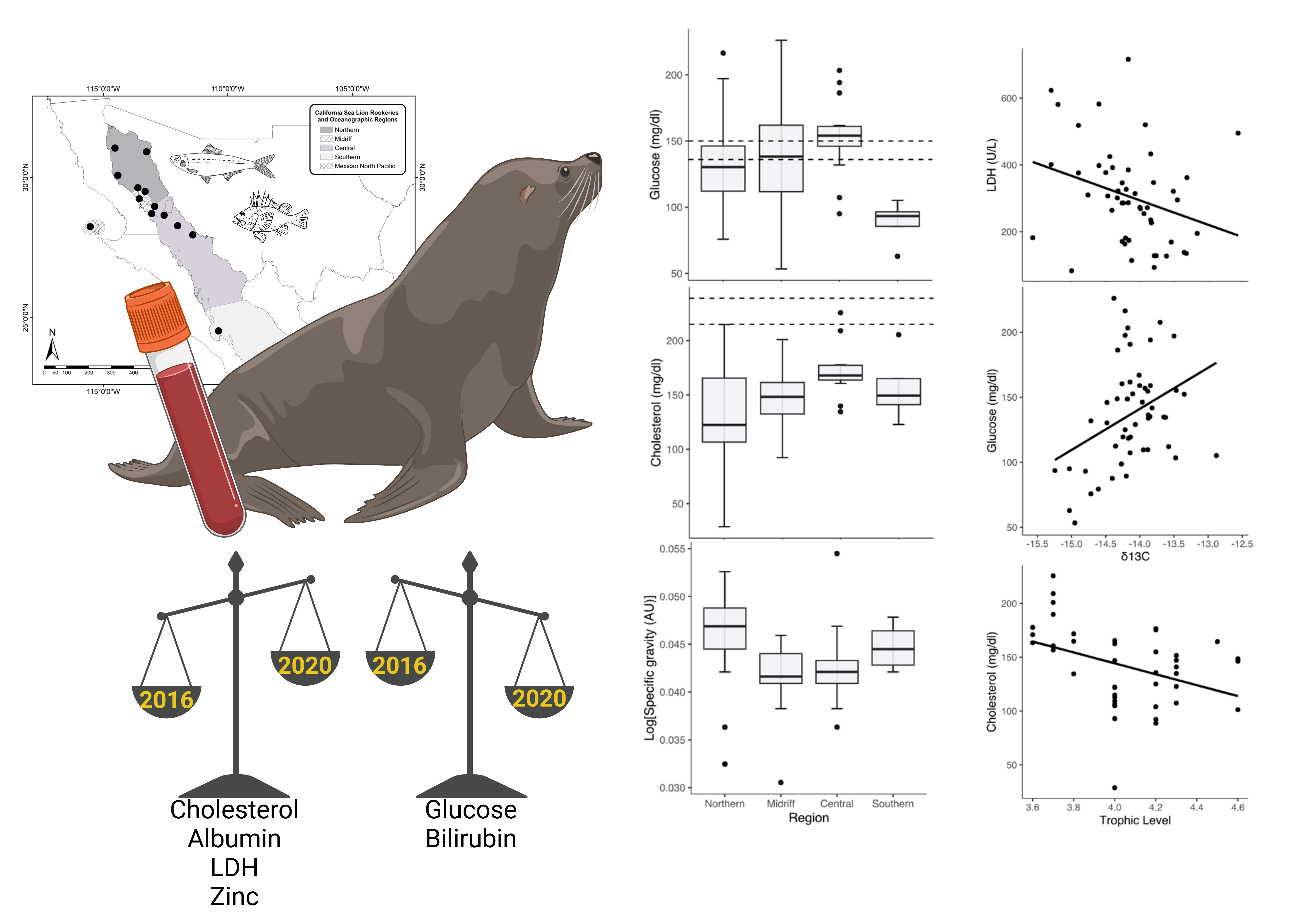

Supplement: Web_Material_coaf056 [file web_material_coaf056.zip › Graphical Abstract Montesinos-Laffont (1).png]
